# REAL-WORLD EFFECTIVENESS ENHANCED INFLUENZA VACCINES IN OLDER ADULTS DURING THREE CONSECUTIVE INFLUENZA SEASONS: 2017–2020

**DOI:** 10.1093/geroni/igac059.1800

**Published:** 2022-12-20

**Authors:** Ian McGovern

**Affiliations:** Seqirus (A CSL Company), Summit, New Jersey, United States

## Abstract

Unenhanced influenza vaccines often fail to elicit adequate immune responses in adults ≥65 years due to immunosenescence. Enhanced influenza vaccines, MF59-adjuvanted trivalent inactivated influenza vaccine (aTIV) and high-dose vaccine (HD-TIV), were specifically developed to overcome this problem. The relative vaccine effectiveness (rVE) of aTIV vs HD-TIV and standard, egg-derived quadrivalent influenza vaccines (QIVe) was estimated in 3 retrospective cohort studies during the 2017-2020 US influenza seasons. Influenza-related medical encounters (IRMEs) were identified using diagnostic codes specific to influenza disease (ICD J09*-J11*) from a dataset that integrated electronic primary care medical records with pharmacy and medical claims. rVE was estimated using propensity score methods adjusting for variables including age, sex, race, ethnicity, geographic location, week of vaccination, frailty and health status. Subgroup analyses included specific age groups and those with high-risk medical conditions. aTIV demonstrated a consistent benefit over QIVe and HD-TIV across all three seasons in the overall study populations, and greater rVE versus QIVe was consistently observed over all three seasons in all age subgroups as well (65-74, 75-84, 85+). aIIV3 was comparable to HD-TIV and more effective than QIVe in a subgroup with high-risk medical conditions. These findings lend further support to the use of aTIV to prevent influenza-related medical encounters in older individuals.

